# Yoga and Mindfulness Interventions for Preschool-Aged Children in Educational Settings: A Systematic Review

**DOI:** 10.3390/ijerph18116091

**Published:** 2021-06-05

**Authors:** Yaoyao Sun, Renee Lamoreau, Samantha O’Connell, Raquel Horlick, Alessandra N. Bazzano

**Affiliations:** 1 School of Nursing and Rehabilitation, Cheeloo College of Medicine, Shandong University, Jinan 250012, China; seinyoyo@gmail.com; 2 School of Science and Engineering, Tulane University, New Orleans, LA 70118, USA; rlamoreau@tulane.edu (R.L.); rhorlick@tulane.edu (R.H.); 3 School of Public Health and Tropical Medicine, Tulane University, New Orleans, LA 70112, USA; soconne1@tulane.edu

**Keywords:** early childhood, school psychology, social-emotional learning, executive function, meditation, self-regulation

## Abstract

Early childhood and the pre-school stage of development constitute a dynamic period for acquisition of social-emotional competencies. Yoga and mindfulness practices (YMP) have become increasingly used in schools for social emotional learning, but less is known about their utility in early childhood settings. A systematic review using PRISMA guidelines was undertaken to explore the effect of YMP on social emotional function among preschool-aged children (3–5 years). The review resulted in identification of 1115 records, of which 80 full text articles were screened, with final inclusion of 16 studies. Included studies evaluated the effect of YMP on social-emotional functioning, and identified the potential for YMP to improve regulatory skills such as behavioral self-regulation and executive function. Among studies reviewed, 13 reported improvements in these domains, but quality appraisal indicated significant variability in risk of bias across studies, and heterogeneity of outcome measurements hindered comparison. Programs appeared to produce better results when implemented for at least 6 weeks and among children who had lower baseline social-emotional functioning. YMP constitute a promising strategy for social emotional development in early childhood settings, but additional rigorously designed studies are needed to expand understanding of how and why these programs are effective.

## 1. Introduction

Participation in yoga and mindfulness meditation has increased over the past decade among both adults and youth in the United States [1,2]. According to data from the National Health Interview Survey, yoga (in the form of physical postures as exercise) was the most commonly used complementary health approach among U.S. adults in 2012 and in 2017, rising from a reported 10% of participants to 14% over the five year period [1].

The second most commonly used complementary health approach was meditation; the use of meditation increased more than threefold from 4% in 2012 to 14% in 2017. The most popular form of meditation for health in the United States is mindfulness meditation, which was introduced in 1982 at the University of Massachusetts Medical Center in the form of Mindfulness Based Stress Reduction (MBSR). Mindfulness meditation was initially defined in MBSR as intentional self-regulation of attention from moment to moment, and other definitions have since emerged centering on focal awareness of experience in the present moment [3]. As adults have increasingly turned to yoga and meditation to improve their health, the percentage of children participating in yoga in the United States also increased significantly between 2012 and 2017 from 3% to 8% [2].

Some evidence on these practices has also become available through peer-reviewed journal articles from research studies of youth-focused interventions. In 2019 alone, 164 journal articles were published on the topic of mindfulness with youth, compared with 11 journal articles on the topic having been published in 2009, just 10 years earlier [4]. Yoga and mindfulness for children are typically presented without reference to spiritual, religious, or historical lineages (e.g., Buddhism, Hatha, etc.) in order to be developmentally appropriate and acceptable in publicly funded settings such as schools. The explosion in popularity has prompted concern that excitement and demand for these programs may be outpacing evidence that they benefit health in youth populations.

Existing reviews of the literature on yoga and mindfulness among youth have examined yoga in schools [5,6] or have looked generally at yoga and mindfulness [7], often with a focus on care for children diagnosed with attention deficit hyperactivity disorder [8] or anxiety [9]. A widely cited study of mindfulness-based interventions in schools was performed in 2012 [10]. Since then, other authors have published systematic reviews of mindfulness with children with autism spectrum disorder [11] and with attention-deficit/hyperactivity disorder (ADHD) [12]. In 2018, a systematic review was published focusing on the utility of mindfulness-based interventions for attention and executive function in children and adolescents [13].

Less is known about the impact of YMP programs among preschool-aged children. Recent work has assessed the impact of yoga and mindfulness on emotional and psychosocial wellbeing among elementary school children [14] and children at Kindergarten [15], but research on younger children participating in YMP is scarce. To date, there are no systematic reviews or meta-analyses examining yoga or mindfulness in early childhood, despite increasing interest in their use among children 3–5 years old [16].

The preschool stage represents a critical period for the development of foundational social emotional and self-regulatory abilities. Self-regulation refers to the internal processes that allow children to manage their thoughts, behavior, and emotions [17]. When children enter school for the first time, early self-regulation is linked with literacy, math, vocabulary, and adaptive classroom behaviors [18,19]. Children with stronger self-regulation skills are better able to manage stress and socialize with peers and teachers [20,21]. Early self-regulation is also linked with important long-term outcomes in adolescence and adulthood. A prospective birth cohort study of 34,323 children in Canada found that more than 40% of 5-year-old children entered the school system with comparative vulnerabilities social-emotional functions associated with early-onset mental health conditions in older age ranges [22]. Another study conducted in 2011 found that childhood self-control was predictive of physical health, substance abuse, financial security, and criminal activity in a large-scale longitudinal study following children over a 30-year period [23].

With the promise and enthusiasm for YMP among children, there is also a need to critically examine the evidence base. This effort serves to inform interested stakeholders and the public, to promote high quality and rigorous research in this area, and to reduce misinformation about benefits, risks and utility of YMP for young children [24]. Understanding the effectiveness of these practices also involves unique conceptual and methodological challenges [25], making interpretation of results more complicated and underscoring the importance of attending to the quality and rigor of results reported. The current systematic review examines yoga and mindfulness interventions in relation to preschool children’s social-emotional outcomes.

## 2. Materials and Methods

The systematic review was conducted in accordance with PRISMA guidelines and a PRIMSA flowchart is included. The study protocol for this review was registered in the PROSPERO database [26] [https://www.crd.york.ac.uk/PROSPERO/] accessed on 21 August 2020, with the registration number CRD42020200206.

### 2.1. Information Sources and Search Strategy

Five databases (PubMed (MEDLINE), EMBASE (Elsevier), PsycInfo (EBSCO), ERIC (EBSCO), and Cochrane Central Register of Controlled Trials) were searched from inception to April 2020. The search consisted of the following terms as Medical Subject Headings (MeSH) and keywords appropriate to each database: “yoga”, “mindfulness”, “meditation”, “child”, “preschool”, “childcare”, “schools”, and “nursery”. Reference lists from relevant review articles and systematic reviews were hand searched to identify additional publications. The American Mindfulness Research Association collection was also searched to further identify key subject area articles. No limits were applied on date, language, or publication status. All articles were accessible within the home library of the research team, Tulane University Libraries databases.

Example search strategy in PubMed: (“Yoga”[MeSH Terms] OR “yoga”[Title/Abstract] OR “Mindfulness”[MeSH Terms] OR “mindful*”[Title/Abstract] OR “self-compassion”[Title/Abstract] OR “Meditation”[MeSH Terms] OR “meditat*”[Title/Abstract] OR “contemplative”[Title/Abstract] OR “contemplation*”[Title/Abstract]) AND (((((“child, preschool”[MeSH Terms] OR “preschool*”[Title/Abstract] OR “pre-school*”[Title/Abstract]) OR (“early child*”[Title/Abstract])) OR (“kindergar*”[Title/Abstract])) OR (“Child Care”[MeSH Terms] OR “Child Care”[Title/Abstract] OR “children care”[Title/Abstract] OR “child day care”[Title/Abstract] OR “children day care”[Title/Abstract] OR “child daycare”[Title/Abstract])) OR (“schools, nursery”[MeSH Terms] OR “nursery school*”[Title/Abstract])). The specific search strategy sample can be found in Supplementary Materials S1.

### 2.2. Eligibility Criteria

The review included yoga and mindfulness studies conducted in early childhood school settings, aimed at improving children’s social emotional development. Only English language studies were included. Where yoga or mindfulness was one component of a complex intervention or a complementary component, half or more of the content was required to be related to mindfulness or yoga. The intervention must have been delivered to children rather than to parents and or caregivers alone. For outcome, studies needed to report at least one child-level social-emotional skill, behavior, or symptom. The definition of “social-emotional” was intentionally broad to encompass a wide variety of skills and behaviors. According to the Collaborative for Academic, Social, and Emotional Learning (CASEL), there are five broad areas of social-emotional competence: self-awareness, self-management, social awareness, relationship skills, and responsible decision-making [27]. Studies that measured at least one of these domains were included in the review.

Intervention studies were eligible if they were randomized controlled trials (RCTs), quasi experimental design trials (QEDs), pre-post-test designs, or otherwise used widely accepted and validated measurement and evaluation methods with statistically appropriate techniques to assess the effectiveness of intervention. The comparison groups in RCTs and QEDs included wait-list control, treatment-as-usual, or other alternative interventions. Pre-post study designs were included if the pre-post-comparison was completed. Participants in included studies were children between 3–5 years old. If a study enrolled children with an overlapping age range (e.g., 2–7 years old), the study was included if the mean age of child participants was less than six years old. Studies that enrolled children with developmental disorders, including intellectual disabilities and autism spectrum disorder, were included if the intervention was provided in a general education setting. Studies were excluded if they were conducted in a special education facility or self-contained classroom, or if they failed to provide information on participant ages.

### 2.3. Study Selection

**Figure 1:**
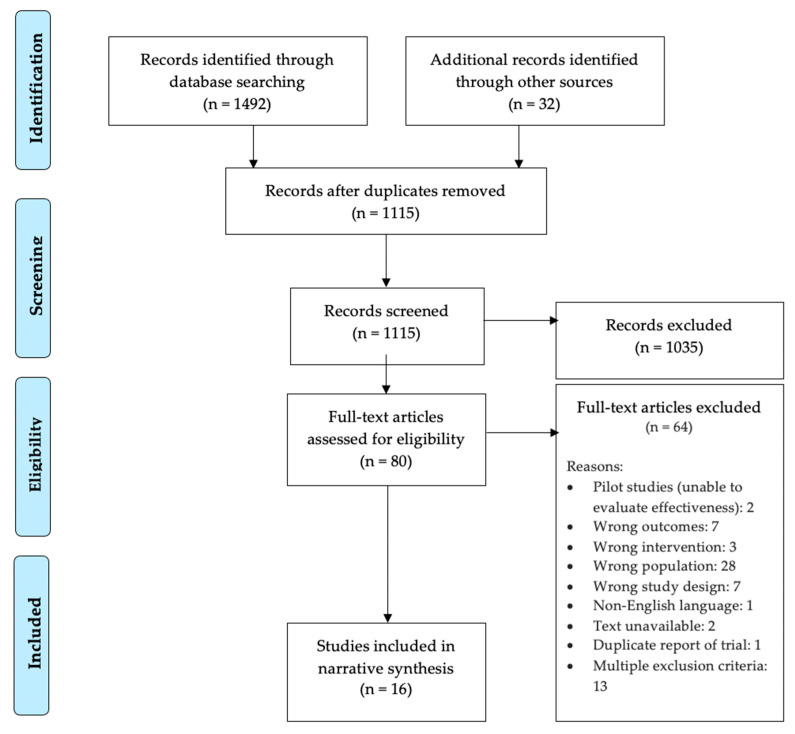
Prisma Flow Chart.

Two authors independently screened studies using prespecified inclusion and exclusion criteria. Titles and abstracts were reviewed and those deemed ineligible were excluded. Articles that met eligibility criteria upon title and abstract review were retrieved and reviewed in depth. Full-text articles were then screened for inclusion and exclusion. The study selection process and reasons for full-text exclusion are shown in Figure 1. Discrepancies were resolved through discussion, and where needed, input from a third author.

### 2.4. Data Abstraction

A standard data abstraction form was adopted from the Cochrane Collaboration [28] to collect all information. An abstraction form was tested by two authors independently using five studies. Concerns with the data abstraction form were resolved by discussion. Data were then abstracted by three authors independently and in duplicate. Results from duplicate data collection were compared and discrepancies were resolved by discussion and consensus. All data were abstracted using Covidence software [29].

The following information was obtained from each study: citation information (e.g., author, year, country of publication, journal), methods (e.g., design, setting, follow-up) participants (e.g., age, gender, race/ethnicity), interventions (e.g., yoga or mindfulness components, timing, delivery, comparison, providers), outcomes (e.g., outcome definitions, time points measured, evaluation methods, imputation of missing data), data analysis (e.g., mean changes reported, missing participants, significant correlations, statistical methods used), and general information (e.g., study conclusions, recommendations, limitations, funding sources, possible conflicts of interest).

### 2.5. Quality Appraisal

Three review authors independently assessed risk of bias for each study using risk of bias tools from Cochrane. The ROBINS-I assessment tool [30] and the RoB 2 risk of bias tool [31] were used to evaluate the quality of non-randomized (*n* = 6) and randomized trials (*n* = 10), respectively. For both tools, “Low risk” correspond to the risk of bias in a high quality study. Disagreements in risk of bias were resolved by discussion and consensus. Both tools include domain items relating to deviations from the intended interventions, missing outcome data, measurement of the outcome, and selection of the reported result. The ROBINS-I also includes items relating to confounding, selection of participants, and classification of intervention. The RoB 2 includes items relating to the randomization process. Risk of bias was assessed for each domain and pooled for an overall risk of bias rating for each study. All papers provided enough information to produce a final rating.

### 2.6. Data Synthesis

Data was synthesized by a tabulated and narrative summary of included studies. Demographic and descriptive information including participants, methodology, interventions, outcome measures, follow up, statistical significance, effect sizes, conclusions, and recommendations of intervention studies were synthesized.

Where sufficient detail was available from the selected studies, information on sub-topics of interest has been presented in addition to an overall synthesis. For example, information is presented on the following: participant characteristics (e.g., gender, developmental disorders), intervention content (entirely yoga, mindfulness intervention and yoga, mindfulness or yoga combined with other interventions), intervention duration, evaluation method, domains of social-emotional development (e.g., emotion regulation, prosocial behavior), and study design (randomized/non-randomized).

## 3. Results

### 3.1. Search Results

The flow diagram of search results is shown in Figure 1. The research team identified 1492 unique records by searching PubMed, Embase, PsycInfo, ERIC and Cochrane Central Register of Controlled Trials and by hand-searching the online bibliography of the American Mindfulness Research Association (AMRA). An additional 32 records were identified through reference lists from relevant review articles and systematic reviews. Of the 1115 records screened after removing duplicates, 80 full-text articles were assessed for eligibility. A total of 17 studies underwent data extraction, and 16 separate trials [15,32,33,34,35,36,37,38,39,40,41,42,43,44,45,46] were eventually included in the final systematic review described below.

### 3.2. Characteristics of Included Studies

Study characteristics are listed in Table 1. Almost all included papers were published in 2015 or later (*n* = 15) [15,32,33,34,35,36,37,38,39,40,42,43,44,45,46] and in peer-reviewed journals (*n* = 14) [15,33,34,35,36,37,38,39,40,42,43,44,45,46]. The journals in which articles were published included Mindfulness (*n* = 4) [36,38,43,46], Journal of Child and Family Studies (*n* = 4) [35,39,40,44], Frontiers in Psychology (*n* = 2) [15,45], Developmental Psychology (*n* = 1) [34], Journal of Developmental and Behavioral Pediatrics (*n* = 1) [33], Journal of Counseling and Development (*n* = 1) [37], and Early Education and Development (*n* = 1) [42]. Two included studies were accessed through published doctoral dissertations available online through ProQuest [32,41].

The included studies utilized a variety of study designs, with the most common being a randomized controlled trial (RCT). A total of 10 trials used an RCT design [15,33,34,35,36,37,40,44,45,46], 5 trials used quasi-experimental design (QED) [32,38,39,42,43] and 1 trial used a pre-post design [41]. Of the 10 RCT trials, half (*n* = 5) were parallel RCTs [15,33,37,45,46] and half (*n* = 5) were cluster RCTs [34,35,36,40,44]. Six studies used wait-list control group [33,34,38,40,43,44] and four used “treatment as usual” control groups [36,37,39,46]. The remaining six studies either used multiple control groups (*n* = 2) [15,45], implemented an alternative control intervention without yoga/mindfulness content (*n* = 2) [32,35], or examined a previous cohort (*n* = 1) [42] or unspecified nonrandomized control group (*n* = 1) [41].

This review features studies from multiple countries and early childhood settings. More than half (*n* = 11) of the trials were conducted in the United States [32,33,34,35,37,39,40,41,42,43,45]. The other five trials were conducted in Singapore [46], Korea [36], Tunisia [15], Canada [44], and Spain [38]. Ten trials [15,32,34,36,39,41,42,43,44,45] were conducted in public elementary schools either in preschool or Kindergarten classrooms. Four trials [35,37,40,46] were conducted in center-based preschool programs (including Head Start centers), which included any freestanding early education centers not connected to an elementary school either publicly or privately financed, and one in a community-based home daycare [33]. One study [38] did not report the setting but enrolled participants from the target age population.

### 3.3. Participants

Across the studies reviewed, sample sizes ranged from 23 to 325 and the cumulative number of children included in all studies was 3584. Mean age of children included in the reviewed literature spanned between 3 years and 5.4 years old. All studies included children of all genders. Nine studies [15,34,37,39,40,43,44,45,46] published information regarding the socioeconomic status (SES) of study participants. Of those that reported on participant SES, participants in four trials [37,43,44,46] were described as low-income or otherwise socioeconomically disadvantaged. Measures of socioeconomic disadvantage included eligibility for free and reduced lunch [43], family monthly household income in comparison with the national median level in Singapore [46], family income in relation to the U.S. federal poverty line [37], and Canadian Learning Opportunities Index (LOI) school district rankings [44]. Information about SES for the other seven included studies is unknown.

Twelve studies [32,34,36,37,39,40,41,42,43,45,46] provided information about race and ethnicity in their samples. A detailed breakdown of participant characteristics is provided in Table 1. Four studies [32,34,39,41] included samples where the majority (at least 50%) of children were non-Hispanic White. The remaining studies included samples where the majority of children were Black/African American (*n* = 2) [40,43], Hispanic (*n* = 3) [37,42,45], or Asian (*n* = 2) [36,46]. One study [33] recruited a sample with an almost equal number of White and Black/African American children.

As described earlier, studies that enrolled children with developmental disorders, including intellectual disabilities and autism spectrum disorder, were included only if they were conducted in an integrated or general education setting. Only three studies [32,33,41] reported on the prevalence of developmental disorders within their general education samples. One study mentioned recruitment of participants with ADHD symptoms but without a formal diagnosis [33]. Another study reported almost 40% of the students included as participants had received services for developmental delay through the special education system [41]. Finally, another study reported that a small proportion of participants had a developmental delay, but the exact percentage was not provided [32].

### 3.4. Mindfulness and Yoga Interventions

The included studies included mindfulness, yoga, or both, and some interventions had additional social-emotional learning components. Five studies [38,42,43,44,46] examined interventions comprised solely of mindfulness, encouraging children to become more aware of physical sensations, thoughts, and feelings. Three studies [15,33,41] examined yoga interventions and taught children physical yoga postures and/or breathing techniques. Two studies [39,40] combined mindfulness and yoga intervention. Lastly, six studies [32,34,35,36,37,45] were “complex” interventions with additional social-emotional learning components. These interventions embedded yoga and/or mindfulness practices within a larger SEL curriculum or provided supplementary instruction. More specifically, complex interventions offered additional instruction in gratitude, kindness, and empathy (*n* = 4) [34,35,36,37], self-reflection (*n* = 1) [45], or emotion identification using picture books (*n* = 1) [32].

Data on program implementation varied widely between the included papers. Six interventions were delivered by a certified yoga teacher or mindfulness instructor [15,33,34,39,40,41], seven by a classroom teacher [32,35,36,38,42,43,45], and three by a researcher [37,44,46]. All interventions had an in-person format, delivered either in a whole-class format (*n* = 12) [15,32,34,35,36,37,38,40,41,42,43,44], small group (*n* = 2) [33,45], individually (*n* = 1) [46], or in an unspecified arrangement (*n* = 1) [39]. Information about class size was not available in any of the papers. The duration of implementation ranged in length from 15 min to one school year. When information was provided about session length, sessions were described as lasting between 10 and 40 min.

### 3.5. Outcome Measurement

Included studies (*n* = 15) [15,32,33,34,35,36,37,38,39,40,42,43,44,45,46] examined either executive function, self-regulation, or a related construct (i.e., attention) as an outcome variable. Six studies [34,35,39,42,43,45] examined executive function (also referred to as effortful control, attentional control, or attention regulation). Seven studies [34,35,37,39,40,44,45] examined self-regulation, a more global construct that encompasses how children control their thoughts, feelings, and behaviors; two studies [32,36] specifically examined emotion regulation. Six studies [15,33,38,39,40,46] examined children’s attentional capacity and four studies [15,33,41,44] examined risk for Attention Deficit and Hyperactivity Disorder (ADHD).

Some of the included studies also examined other domains of social-emotional functioning beyond executive function and self-regulation. Seven studies [33,34,36,37,43,44,45] specifically examined prosocial behaviors or changes to theory of mind and empathy. Five studies [32,36,38,39,45] examined broader indicators of positive social-emotional development like resilience, psychological well-being, and psychosocial adjustment. Studies used a combination of teacher report and/or direct child assessment, often reporting results from both sources in the published reports.

### 3.6. Quality Appraisal

The overall ratings indicated significant variability in risk of bias across the sample. For the ten RCT studies [15,33,34,35,36,37,40,44,45,46] evaluated with the RoB 2, four [15,34,40,45] had some concerns, and six [33,35,36,37,44,46] had a high risk of bias. For the six [32,38,39,41,42,43] non-RCT studies evaluated with the ROBINS-I, one study [41] had a critical risk of bias, two [32,42] had a serious risk of bias, and three [38,39,43] had a moderate risk of bias. None of the studies were found to have low risk of bias in all domains. The table summarizing quality appraisal is provided in Appendix A Table A1 and Table A2. Graphs of risk of bias for RCT and non-RCT studies are provided in Figures S1 and S2, and summaries for risk of bias are provided in Figures S3 and S4.

The source of bias differed between RCT and non-RCT studies. Specifically, for the RCT studies, bias in outcome measurement was the most frequent source of high risk of bias (*n* = 4) [33,35,36,44], followed by bias due to deviations from intended interventions (*n* = 3) [33,37,46], bias arising from the randomization process (*n* = 2) [36,44], and bias due to missing outcome data (*n* = 1) [46]. For the non-RCT studies, the critical risk of bias came from bias due to deviations from intended interventions [41]. The bias in measurement of outcomes was the most frequent serious risk of bias (*n* = 3) [32,41,42] followed by bias due to missing data (*n* = 2) [32,41].

### 3.7. Research Question 1: What Available Evidence Suggests that Yoga and Mindfulness Interventions Improve Social Emotional Outcomes and Cognitive or Executive Function for Preschool-Aged Children?

Table 2 summarizes the results by study. Almost all (*n* = 13) studies [15,33,34,35,36,37,38,39,40,42,43,44,45] reported that yoga and mindfulness programs improved at least one social emotional outcome in preschool-aged children. The included studies examined multiple social-emotional domains, namely self-regulation, executive function, and attention. Three studies [32,41,46] failed to find evidence of a significant intervention effect; however, it is important to note that two of the three studies [32,41] were unpublished dissertations.

#### 3.7.1. Behavioral Self-Regulation

Self-regulation refers to processes that allow children to manage their thoughts, behavior, and emotions [17]. Seven studies [34,35,37,39,40,44,45] reported intervention effects on behavioral self-regulation. To measure behavioral self-regulation, the most commonly used measure was the Head-Toes-Knees-Shoulders task (HTKS; [47]), a validated measure of self-regulation typically administered to children 4–8 years old. This task requires children to perform the opposite action of a behavioral command (e.g., touch toes when told by examiner to touch head). This requires children to inhibit the dominant or automatic response of imitating the examiner. Of the five studies [35,39,40,44,45] that used the HTKS as an outcome measure, children who participated in mindfulness and yoga interventions outperformed a control group. These results suggest that yoga and mindfulness interventions may have a favorable impact on the behavioral aspects of self-regulation as measured by the HTKS.

Other measures used to measure behavioral self-regulation included the Delay of Gratification Task [48], the Toy Wrap/Wait Task [49], and the Child Observation Mindfulness Measure (C-OMM [50]). Using these other measures, researchers found more inconsistent results. The Delay of Gratification Task requires children to choose between having a smaller reward immediately or a larger reward later. Flook et al., 2015 [34], failed to find a significant difference between the intervention groups pre- and post-test on the Delay of Gratification Task. Similar to the Delay of Gratification Task, the Toy Wrap Task requires children to wait for a surprise while the examiner “wraps” it. The study conducted in 2015 [39] found a significant main effect of the intervention on Toy Wrap, but only a trending main effect of the intervention on Toy Wait. Lastly, the C-OMM is an observational measure used to assess children’s self-regulated attention and orientation to experience. A study from 2018 [37] did not find significant intervention effects on the C-OMM but noted that descriptive statistics were trending in favor of the intervention group.

#### 3.7.2. Emotion Regulation

Under the umbrella of self-regulation, emotion regulation describes how children manage affective states [51]. Two studies [32,36] examined child emotion regulation using the Emotion Regulation Checklist (ERC; [52]). These two studies found conflicting results. The 2019 study [32] failed to see an effect of time or of intervention on either teacher- or parent-reported ERC scores. While the study conducted in 2020 [36] reported that the intervention group began to show significantly higher levels of emotional regulation than the control group, the difference was only significant at later timepoints.

#### 3.7.3. Executive Function

Six studies [34,35,39,42,43,45] measured children’s executive function (EF), a construct closely related to self-regulation. Executive function underlies goal-directed behavior and encompasses (1) working memory, (2) inhibitory control, and (3) set shifting/flexibility [53]. The measures used to assess executive function varied widely by study and included the Behavior Rating Inventory of Executive Function–Preschool (BREIF-P) (*n* = 2) [35,42], Peg/Pencil Tapping (*n* = 2) [39,45], Flanker Task (*n* = 2) [34,43], Minnesota Executive Function Scale (MEFS) (*n* = 1) [45], Go/No-Go (*n* = 1) [35], Dimensional Change Card Sort Task (DCCS) (*n* = 1) [34], and the Hearts and Flowers Task (*n* = 1) [43].

Overall, the effect of yoga/mindfulness participation on children’s executive function was mixed. For example, using the BREIF-P, a teacher-rated questionnaire of children’s executive function, Thierry et al., 2016 [42] found positive impacts on teacher-reported working memory and planning/organizing. In comparison, Jackman et al., 2019 [35] found that the intervention group showed decreased cognitive flexibility post-test as measured on the BRIEF-P compared to a comparison group.

Similar inconsistencies emerged in the remaining studies that used other EF measures. For the Flanker task, only one [43] found a significant main effect of the mindfulness program. For Pencil/Peg tapping, only one [39] found a significant main effect of the mindful yoga intervention. Post-test scores on the Minnesota Executive Function Scale (MEFS), Go/No-Go, and Dimensional Change Card Sort Task (DCCS) did not differ between intervention and control groups in the studies that used these measures. The study by Thierry et al. [43], however, found significant intervention effects on reaction time in the Hearts and Flowers Task.

#### 3.7.4. Attentional Capacities

Six studies [15,33,38,39,40,46] assessed attentional capacities, including visual attention, attention regulation, and attentional control. Each study used a different measure to assess at least one dimension of attention. All of the measures were direct child assessments, including the Developmental Neuropsychological Assessment (NEPSY; [54]), Attention Sustained task (AST; [55]), Shortened Child Attention Network Task (ANT; [56]), KiTAP Test of Attentional Performance for Children [57], and the Children Neuropsychological Maturity Questionnaire (CUMANIN; [58]). Five of the six studies [15,38,39,40,46] found that participation in yoga and mindfulness interventions was associated with improvements in one or more attentional capacities.

#### 3.7.5. ADHD Symptoms

Four studies [15,33,41,44] looked specifically at attention-deficit/hyperactivity disorder (ADHD) symptoms using the Conners Teacher Rating Scale‚ Revised: Short Form (CTRS-R:S; [59]), ADHD Rating Scale-IV [60], and the Strengths and Difficulties Questionnaire (SDQ; [61]). ADHD is a neurodevelopmental disorder characterized by deficits in executive function and difficulties with concentration and impulsivity. One study [41] found that participation in a yoga intervention did not have a significant effect on oppositional behavior or inattentive symptoms (as measured on the CTRS-R:S) but did have an effect on a global ADHD index. Using the ADHD RS-IV, two studies ([33] and [15]) found that intervention participation was associated with improvements in both hyperactive and inattentive behavior. Two additional studies ([44] and [33]) both reported observed improvements on the hyperactive-inattentive scale of the Strengths and Difficulties Questionnaire.

#### 3.7.6. Peer and Prosocial Behavior

Seven studies [33,34,36,37,43,44,45] reported on peer and prosocial behavior in relation to participation in yoga/mindfulness interventions. There was no consistency in measurement across studies: each study used a different measure to assess peer and prosocial behavior. Measures included the inCLASS (Individualized Classroom Assessment Scoring System), Theory of Mind Scale, Social Skills Improvement System-Rating Scales (SSIS-RS), Sharing Task, Modified Professional Behavioral Questionnaire (Mod-PBQ), Teacher-Rated Social Competence (TSC), and Strengths and Difficulties Questionnaire (SDQ). Results again were mixed, with no clear consensus across the seven studies. Four studies found significant effects of yoga/mindfulness programs on peer and prosocial behaviors, and three failed to find significant effects.

#### 3.7.7. General Indicators of Social-Emotional Functioning

Five studies [32,36,38,39,45] reported on more general indicators of social-emotional functioning like resilience, psychosocial adjustment, and broad problem behaviors. Measures included the Devereux Early Childhood Assessment for Preschoolers (DECA-P2), Behavioral Assessment System for Children (BASC-2), Korean Personality Rating Scale for Children (KPRC), Child Behavior Questionnaire (CBQ), and Child Behavior Rating Scale (CBRS). A 2019 study [38] found that participation in the MindKinder program was associated with reductions in externalizing behaviors (e.g., aggression) and general behavior problems. A study from 2020 [36] reported that children who participated in a mindfulness-based intervention had higher resilience scores post-test. Higher resilience scores indicated that children had better coping skills and were more flexible and responsive to the environment. The remaining studies (*n* = 3) [32,39,45] reported null results for these general social-emotional measures.

### 3.8. Research Question 2: How Does the Effectiveness of Yoga and Mindfulness Programs Differ by Population, Duration of Intervention, and Program Content?

#### 3.8.1. Population

Study results were consistent across different demographic groups. Interventions were effective across a broad range of participant groups representing children of diverse racial/ethnic backgrounds and SES levels. However, only two studies [41,45] explicitly examined participant demographic characteristics as potential moderators of program effect. A 2010 study [41] reported that gender moderated the effect of yoga intervention, such that girls demonstrated higher post-test attention scores after participating in the yoga program where boys did not. In addition, a 2018 [45] study drew from two cohorts of children, one of which was largely (97%) Hispanic located in Houston, TX, and another that was entirely (100%) Black/African-American located in Washington, DC. Children at the Houston site showed larger improvements on executive function measures than children at the DC site. However, differences in engagement by location, rather than responsiveness to the intervention or cultural competency, were cited by the authors.

#### 3.8.2. Duration of Intervention

Duration (i.e., how long children received the yoga/mindfulness program) appeared to potentially influence program effectiveness. When comparing the studies that had null results (*n* = 3) [32,41,46] to those that had favorable results (*n* = 13) [15,33,34,35,36,37,38,39,40,42,43,44,45], program length differed. Of the three studies that had null results, children’s participation ranged from 15 min to 4 weeks. For the remaining 13 studies reporting favorable results, children participated in interventions for comparatively longer periods of time (i.e., at least six weeks).

#### 3.8.3. Frequency of Intervention

Frequency (i.e., how often children received the yoga/mindfulness program) differed widely across studies, making it difficult to draw conclusions about frequency as a potential moderator. Programs were implemented on a daily basis (*n* = 6) [35,36,39,42,43,45], twice a week (*n* = 5) [15,33,34,40,41], three times a week (*n* = 2) [32,44], four times a week (*n* = 1) [37], or six times a week (*n* = 1) [38]. One study [46] evaluated a single-session intervention.

#### 3.8.4. Other Moderators

Studies among children with lower baseline social-emotional functioning described the largest increase in social emotional function from yoga/mindfulness interventions. A 2015 study [34] found that children in the mindfulness group with lower baseline levels of social competence and executive functioning showed larger growth in social competence over time. Similarly, another study from the same year [39] reported that children who were most at risk of self-regulation dysfunction benefited the most from the mindful yoga intervention. A 2018 study [44] found that mindfulness-based programs are particularly effective for children with difficulties in related areas (e.g., self-regulation, prosocial behavior, and hyperactivity). Lastly, another study [33] reported that children with more significant ADHD symptoms at baseline show more dramatic improvements in hyperactivity and inattention after practicing yoga.

## 4. Discussion

This systematic review is the first to assess the effects of YMP on social emotional outcomes in preschool-aged children. Our results align with those of previous systematic reviews and meta-analyses [12,62,63] indicating the promise for positive effects from YMP for children aged 3–5 years, tempered by caution regarding the level of evidence available. Among the results, 13 of 16 included studies (81%) reported beneficial effects of yoga/mindfulness on at least one SEL outcome. Specifically, positive effects were found for SEL domains of: behavioral self-regulation, emotion regulation, attentional capacities, executive function, ADHD symptoms, peer and prosocial behavior, and other general indicators of social-emotional functioning. Studies reviewed also indicated that YMP programs can be successfully adapted to meet the unique needs of children in early childhood settings.

Behavioral self-regulation was one of the most frequently studied outcomes, assessed in 7 of 16 (44%) studies. A majority of the studies (71% or 5 of 7 studies) evaluating behavioral self-regulation found an improvement following the intervention. This improvement varied depending on the outcome measurement used. Whereas improvement was found in all studies utilizing the HTKS measure [47], and one study measuring Toy Wrap [49], there were no significant effects found in studies measuring self-regulation by the delay of gratification task [48], the Toy Wait task [49], or the C-OMM [50]. Under the umbrella of self-regulation, emotion regulation was also assessed. With only two studies (13% or 2 of 16 studies) evaluating emotion regulation, the results were mixed. One found a significant positive effect, and one did not.

Executive function, the cognitive processes underlying behavioral self-regulation, was the second most highly studied outcome (38% or 6 of 16 studies). However, considerable heterogeneity in measurement tools and results make it difficult to draw conclusions on the effect of mindfulness and yoga intervention. BRIEF-P [64], Flanker task [65], and Pencil/Peg Tagging [66] were utilized. Inconsistent results were found among the three measures with positive effects and no effects. Studies using other measures did not find significant results on executive functions except one study with Hearts and Flowers Task [67] reporting significant intervention effect on reaction time.

Children between 3–5 years old experience rapid growth and development of regulatory abilities. Interventions delivered in the preschool period may occur during a sensitive period in development where these skills are first coming “online” [68]. Self-regulation in early childhood is important for school readiness and for later academic outcomes [20]. By summarizing the evidence on self-regulation using YMP interventions, results of the review illustrate that behavioral self-regulation was the most targeted outcome and could be improved, with some promising effects on emotion regulation and executive functioning.

Many of the interventions explicitly taught kindness and/or social skills, or hypothesized that participation in YMP would have a downstream effect on peer interactions. Therefore, outcomes related to prosocial behavior were assessed in 7 of 16 (44%) studies. However, measurement tools and effects for prosocial behavior varied across studies. A majority of studies (4 of 7 or 57%) reported a positive impact of yoga/mindfulness interventions. The remaining 3 studies (43%) did not report significant effects. The mixed results here are not surprising given the variety of programs represented in the review. Direct instruction of social skills using role plays and activities may be the best way to teach prosocial behaviors in early childhood classrooms [69].

A number of studies examined attentional capacities (38% or 6 of 16 studies), or deficits in attention by looking at ADHD symptoms (25% or 4 of 16 studies). Most studies (83%) found significant improvements in attentional capacities following intervention. As unique measurement tools were used in each study, this finding did not correspond to measurement type. In regard to ADHD symptoms, all four studies reported at least one significant finding linking yoga/mindfulness participation with reductions in ADHD symptoms. These findings align with prior research suggesting that yoga is a promising intervention for children with attention problems [70]. YMP may enhance body awareness, improve concentration, and promote relaxation, leading to the development of better attentional capacities and a reduction in ADHD symptoms.

It is important to examine what interventions work for different populations and under what conditions. The second research question examined whether results were consistent across populations or dimensions of implementation (e.g., frequency, duration). There were no differences based on population or frequency of intervention delivery. Variation was noted, however, according to duration, or the total length of time that children received the YMP intervention. For interventions lasting from 15 min to 4 weeks, null results were reported. In contrast, interventions lasting at least six weeks reported at least one favorable SEL-related result. Out of all possible dimensions of implementation, duration seemed to matter the most. Children who participate in YMP programs for a longer period of time receive a higher “dosage” of the intervention, and dosage may an important predictor of child outcomes in educational settings [71].

In regard to other moderators of program effect, children with poorer baseline skills showed the most improvement following yoga/mindfulness interventions. This was true in the case of children with lower levels of social competence and executive functioning. [39], for children with difficulties in SEL skills in [44], and with children with more significant ADHD symptoms [33]. This finding aligns with a compensatory hypothesis; children with lower skills may benefit the most from YMP interventions and have more “room to grow” [72].

Future research should continue to investigate how yoga/mindfulness interventions may improve SEL outcomes for other at-risk populations of young children. In the adult literature [73], mindfulness/yoga interventions have demonstrated moderate effects on psychiatric symptoms in trauma-exposed populations. The same might be true for young children; an estimated one in three children from low-income families are exposed to violence before the age of five [74]. Trauma exposure in early childhood is particularly harmful to the developing brain and has lifelong consequences on mental and physical well-being. Stress can “get under the skin” in ways that inhibits behavioral self-regulation and executive function development [75]. Yoga and mindfulness interventions may provide young children with the tools and self-regulatory capacities to counteract some of the adverse effects of early trauma exposure.

The current study has several limitations. Firstly, the success of an intervention hinges on the quality of implementation, but data on implementation was limited. Future studies should provide more in-depth implementation data on acceptability, feasibility, and student engagement. Secondly, quantitative analysis was not conducted in the present study, limited by the diversity of measurement tools in each outcome. This makes it impossible to draw conclusions based on pooled effect size estimates. The review was also unable to determine with confidence if yoga and mindfulness interventions might work best for certain populations of children. This would require future research to conduct moderation analyses of program effects.

Lastly, while review distinguished between different SEL domains, there is substantial overlap between self-regulation and executive function, for example. Constructs were classified based on the language used to describe the measures along with precedent. The results should be interpreted with the understanding that social-emotional functioning often involves multiple, coordinated skills that are difficult to parse apart.

The results of this study can be used to inform YMP programming in early childhood settings. In general, teachers in elementary [76] and early childhood settings [77] have indicated that YMP are feasible and acceptable to implement in educational settings. Education and childcare centers may also choose to integrate yoga and mindfulness practices within existing SEL programs. For example, one study [37] evaluated an intervention that combined SEL and mindfulness, with positive impact on children’s self-regulation. Educators and school staff may find it easier and more acceptable to introduce yoga and/or mindfulness content in the context of existing programming.

The results of this systematic review may also inform educational policy and practice in early childhood settings, as well as contribute to additional rigor and planning of future research on yoga and mindfulness with young children. Given the relatively small number of studies (*n* = 16) included in the review and risk of bias, more research is needed. Future research should continue to investigate the efficacy and effectiveness of YMP interventions in diverse contexts. It is important to determine under what conditions and for whom these interventions are best suited. Our review of the literature indicated that children with the lowest baseline social-emotional skills may benefit the most from YMP interventions. Early childhood centers may use universal screening using an instrument to identify children with lower baseline scores. The quality of implementation matters in health and education prevention programs [78], and future research should continue to assess links between YMP implementation and outcome data.

## 5. Conclusions

Overall, this systematic review provided some evidence that yoga and mindfulness are promising practices for addressing social emotional development among preschool-aged children. Much of the prior work in this area has examined older children or has looked specifically at isolated diagnostic categories (e.g., children with ADHD). The review identified YMP having favorable effects on several regulatory domains, as well as on attentional capacities, peer and prosocial behavior, and general well-being, but due to heterogeneity of measurement of social-emotional outcomes and risk of bias, the level of evidence remains moderate. Additional methodologically rigorous studies are required to assess pooled data and to increase confidence in the level of evidence. As YMP continue to be used in schools, a clearer understanding of how and why it may be beneficial for young children will emerge.

## Figures and Tables

**Table 1:** Descriptive information about study location, setting, and participant characteristics.

Study	Design	Country	Setting	Mean Age (±SD)	Sample Size	Gender	Race/Ethnicity	Other Relevant Sample Characteristics
Thierry et al., 2018 [43]	QED	USA	Public preschools in a large-sized city in the Southwestern U.S.	4.5 years (±0.32)	325	51% Female 49% Male	57% African American, 40% Latina/o, 1% White, 2% Other	Spanish as first language = 27% Free/reduced lunch = 98%
Lim and Qu, 2017 [46]	RCT, parallel	Singapore	Childcare centers in urban city	65.1 months (±0.32)	122	48% Female 52% Male	100% Singaporean	Parents with a high school or above a high school education = 90.7% of mothers and 88.8% of fathers Families with monthly household income below the national median monthly household income = 70.4%
Kim et al., 2020 [36]	RCT, cluster	Korea	Four Korean preschools	3 years (SD missing)	83	54% Male 46% Female	100% Korean	None reported
Zelazo et al., 2018 [45]	RCT, parallel	USA	Two preschools serving low- income families in urban cities (Houston, TX and Washington, DC)	57 months (±0.32)	218	46% Female 54% Male	Houston, TX Cohort: 55% White; 32% More than one, 9% African American, 3% Native American, 97.4% Hispanic Washington, DC Cohort: 100% African American	None reported
Jarraya et al., 2019 [15]	RCT, parallel	Tunisia	Private Tunisian Kindergarten in urban setting	5.2 years (±0.4)	45	62% Female 39% Male	None reported	Participants were from middle class families with a corresponding average to high socio-economic status. Children with a lack of any frequent participation in yoga exercise programs for at least 6 months prior to the study and no daily intake of medication
Cohen et al., 2018 [33]	RCT, parallel	USA	A local urban, community-based preschool	49 months (±9)	23	35% Female 65% Male	43% White, 4% Asian, 39% Black/African American, 9% Mixed, 4% Unknown	Children had four or more ADHD symptoms as rated by teachers or parents on the ADHD Rating Scale-IV Preschool Version; One child had autism spectrum disorder; Two children had an ADHD diagnosis; One child took ADHD medication
Lemberger-Truelove et al., 2018 [37]	RCT, parallel	USA	A childcare facility serving low-income children enrolled in a summer session at a childcare center in the southwestern United States	3.9 years (±0.79)	23	52% Female 48% Male	52% Hispanic, 26% mixed race, 22% white	The sample consisted of children from low- income households, with 35% under the poverty line (i.e., under $24,000 for a four-person household) and another 43% under twice the poverty line (i.e., under $16,000 for a four-person household).
Viglas and Perlman, 2018 [44]	RCT, cluster	Canada	Three public preschools in in the ethnically diverse city of Toronto, Ontario in Canada	62.32 months (±7.5)	127	42% Female 58% Male	None reported	All three schools in this study experienced somewhat higher levels of external challenges (e.g., parents’ education and income, poverty and proportion of lone-parent families as measured by the Toronto District School Board Learning Opportunities Index (LOI).
Razza et al., 2020 [40]	RCT, cluster	USA	An urban Head Start center in a mid-sized city	4.1 years (±0.37)	89	50% Female 50% Male	74% African American 14.3% mixed-race	City residents living below the federal poverty level = 33.33% for all residents, 43% among families with children under age 18; female headed households = 54%; city had highest per capita murder rate in all of New York state in 2013; Rate of post-traumatic stress in community = 51%.
Razza et al., 2015 [39]	QED	USA	Two full-day universal pre-kindergarten classrooms within the same urban public elementary school	51.1 months (±3.8)	34	62% Female 38% Male	52% White, 7% Hispanic, 34% Black or African American, 7% Other	Parents with a master’s or professional degree = 60% married families = 66% cohabiting = 7% average child: adult ratio of 1.6 (SD = 1.1; range 0.3–6.0)
Jackman et al., 2019 [35]	RCT, cluster	USA	Head Start classrooms in Jefferson and Franklin counties	3 years, 8 months (+6 months)	262	51.5% Female 48.5% Male	None reported	None reported
Thierry et al., 2016 [42]	QED	USA	An urban elementary school located in a large-size city	4.55 years (±0.30)	47	49% Female 51% Male	85% Hispanic, 9% African American, 6% White	Students qualified for free or reduced-price lunch = 72% Average family income: Intervention Group = $34,416 Control Group = $31,320
Flook et al., 2015 [34]	RCT, cluster	USA	Six different elementary schools within a public school district in a medium-sized city Seven classrooms from the study schools	4.67 years (±0.27)	68	50% Female 49% Male	58.8% Caucasian, 11.8% Hispanic, 5.9% African American, 10.3% Asian/Pacific Islander, 11.8% Other/mixed ethnicity	Parents with four-year college degree = 72.1% Children considered socioeconomically disadvantaged = 37.9%
Carrozza 2019 [32]	QED	USA	Private Preschool in urban city	IG: 3 years, 5 months old (± 0.294, 3 months) CG: 3 years, 7 months old (±0.487, 5 months)	27	57% Male 43% Female	52% White 5% Asian 10% Hispanic 33% Multi-racial	Autism: 10% (*n* = 2); Typical Development: 81% (*n* = 17) Speech Delay: 10% (*n* = 2); Motor Delay: 10% (*n* = 2)
Rich 2010 [41]	Pre-Post design trial	USA	Public Preschool in two suburban communities	Mean age: 4.63 years (SD missing)	49	59% Female 41% Male	**District A**: 3.0% African American, 2.8% Asian, 1.7% Hispanic, 0.7% Native Hawaiian/Pacific Islander, 0.2% Native American, 91% White, 0.2% Multiracial/Non-Hispanic; **District B**: 14% African American, 3.7% Asian, 4.6% Hispanic, 0.2% Native Hawaiian/Pacific Islander, 0.1% Native American, 75% White, 1.2% Multiracial/Non-Hispanic.	Approximately 40% of the students in the study received services for students with developmental delays from the special education department.
Moreno-Gómez and Cejudo, 2019 [38]	QED	Spain	Kindergarten children obtained through an incidental non-probability sampling method or by accessibility	5.08 years (±0.37)	74	47% Male 53% Female	Unknown	None provided

Note. SD = standard deviation, QED = quasi experimental design, RCT = randomized controlled trial, ADHD = attention-deficit/hyperactivity disorder.

**Table 2:** Intervention Implementation and Results.

Study	Intervention Classification and Description	Comparator	Instructor and Location	Duration (# Sessions if Provided)	Frequency	SEL Outcome Measures	Summary of Conclusions	Overall Risk of Bias
Thierry 2018 [43]	Mindfulness: Five learning units, each unit contained 2–4 lessons (18 lessons total), with extension activities and strategies to enhance students’ self-regulation and self-awareness	Wait-list	Teacher in classroom	One school year	2 weeks per unit incorporated into the school day at teacher’s discretion	Executive function: Flanker Task, Hearts and Flowers Task Prosocial Skills: Social Skills Improvement System-Rating Scales (SSIS-RS)	Students in the mindfulness schools showed greater improvement in executive functions than students in the business-as-usual schools. There were no differences between groups on measures of prosocial behavior.	Moderate *
Lim and Qu 2017 [46]	Mindfulness: Three 5-min activities: 5-min stretching with balance and focusing on the body posture, 5-min listening to the tapping and focusing on the sound, 5-min counting the breath and focusing on the breath	Active control included simple dance, sing and counting guided by researcher	Researcher in quiet unused classroom	15 min	One 15-min session	Attention: Shortened Child Attention Network Task (ANT), Global—Local Test (GLT)	There was no effect of mindfulness training on children’s performance on the ANT.	High
Viglas 2018 [44]	Mindfulness: Lessons on “external” and “internal” experiential mindful awareness practices and lessons on heartfulness (i.e., kindness and caring). Following each lesson, children were asked to write or draw in their mindfulness journals.	Wait-list	Researcher in classroom	6 weeks (18 sessions)	3 times a week, 20 min per session	Behavioral Self-Regulation: Head-Toes-Knees-Shoulders (HTKS) General Social-Emotional Functioning: Strengths and Difficulties Questionnaire (SDQ)	Children in the mindfulness group showed greater improvement in self-regulation, were more prosocial and less hyperactive compared to children in the control group at Time 2.	High
Thierry 2016 [42]	Mindfulness: MindUP program lessons taught over the course of the school year and three times each day, students engaged in core mindfulness practice, deep breathing with a focus on a single resonant sound	Business-as-usual	Teacher in classroom	One school year	Daily	Executive Function: Behavior Rating Inventory of Executive Function-Preschool (BRIEF-P)	At the end of the prekindergarten year, students in the mindfulness program showed improvements in teacher-reported executive function skills, specific ally related to working memory and planning and organizing, whereas children in the business-as-usual group showed a decline in these areas.	Serious *
Moreno-Gomez 2019 [38]	Mindfulness: The program was organized into four content blocks: (1) mindfulness meditation techniques, (2) work with mandalas, (3) visualization techniques, and (4) body awareness	Wait-list	Teacher and Researcher in classroom	6 months (144 total sessions)	Six times a week, 15 min per session	Attention: Children Neuropsychological Maturity Questionnaire (CUMANIN) General Social-Emotional Functioning: Behavioral Assessment System for Children (BASC-2)	There was a significant reduction in the scores of global maladaptive dimensions, behavioral symptoms index and externalized and academic problems, among the experimental group. There was a significant increase in scores for the experimental group in the dimensions of global development, non-verbal development, visual perception, and attention.	Moderate
Jarraya 2019 [15]	Yoga: Adapted Hatha yoga including a 5 min warm up period of jogging and jumping followed by yoga specific stretching and breathing, 15 min yoga postures (Asana), 5 min breathing techniques, ending with yogic games. Throughout different phases, a story was told to motivate the children to actively participate.	Post-test with active (physical education) and passive (no physical activity) control groups	Certified yoga teacher in Kindergarten gym	12 weeks (24 sessions)	Twice per week, 30 min per session	ADHD Symptoms: ADHD Rating Scale-IV Attention: Developmental Neuropsychological Assessment (NEPSY)	In comparison to the active and passive control groups, yoga had a significant positive impact on ADHD symptoms. Yoga had significant positive impact on completion times in two visuomotor precision tasks in comparison to the active control group and on visual attention scores in comparison to the passive control group.	Some concerns
Cohen 2018 [33]	Yoga: Manualized curriculum from If I Was a Bird Yoga, sequence of breathing exercises and poses consistent over the intervention period	Wait-list	Trained children’s yoga instructors in separate room from classroom	6 weeks (12 sessions)	Twice per week, 30 min per session	ADHD symptoms: ADHD Rating Scale-IV, Preschool Version Attention: KiTAP Test of Attentional Performance for Children General Social-Emotional Functioning: Strengths and Difficulties Questionnaire (SDQ)	Children in the yoga group had faster reaction times on the KiTAP Go/No go task, fewer distractibility errors of omission, but more commission errors than children in the control group.	High
Rich 2010 [41]	Yoga: The yoga teacher utilized an instrumental compact disc that was incorporated into the activities in the lesson	Post-test	Certified yoga teacher in classroom	4 weeks (8 total sessions)	Twice a week, 20 min per session	ADHD symptoms: Conners Teacher Rating Scale‚ Revised: Short Form (CTRS-R:S)	The overall findings of the study did not support the hypothesis that preschool students who participate in yoga therapy will demonstrate increased attention to tasks in the classroom.	Critical *
Razza 2020 [40]	Combined Yoga and Mindfulness: Each session began with a centering activity, progressed through a set of child-centered yoga poses, and concluded with a brief relaxation activity	Wait-list	Certified yoga teacher in gym	8 weeks	twice a week, 25 min each 16 sessions	Attention: Attention Sustained task (AST) Behavioral self-regulation: Head-Toes-Knees-Shoulders (HTKS)	Mindfulness and yoga produced significant increases in children’s behavioral and attention regulation.	Some concerns
Razza 2015 [39]	Combined Yoga and Mindfulness: Daily practice included breathing and sun salutations during morning circle, yoga postures linked to literacy activities I the afternoon, and breathing exercises during transition periods.	Post-test	Certified yoga teacher in classroom	25 weeks	Daily, average length of time increased gradually across the school year from 10 min per day to 30 min per day	Attention: AST Behavioral self-regulation: HTKS, Toy Wrap Task, Toy Wait Task Executive function: Pencil Tapping General Social-Emotional Functioning: Children Behavior Questionnaire (CBQ)	Mindful yoga produced significant benefits for young children’s self-regulation.	Moderate *
Zelazo 2018 [45]	Complex: A variety of brief (e.g., 2 min) mindfulness and relaxation practices adapted for children and three EF-challenging games	Business-as-usual	Local teacher recruited from city and trained by researchers in unknown school location	6 weeks (30 small-group sessions)	Daily, 24 min per session	Behavioral self-regulation: HTKS Executive function: Peg tapping, Minnesota Executive Function Scale (MEFS) Prosocial skills: Theory of Mind Scale General social-emotional functioning: Child Behavior Questionnaire (CBQ), Child Behavior Rating Scale (CBRS)	A brief small-group school-based mindfulness and reflection intervention produced significant improvements in executive function at follow-up (4 weeks post-test) compared to business-as-usual.	Some concerns
Kim 2020 [36]	Complex: Key daily practices: 8-min guided Samatha meditation at 10 a.m. and nine daily mindfulness activities: Samatha meditation, loving kindness, yoga, gratitude and interconnection activities, kindness and compassion reported, feelings finder, Super Me, Are You Present for Me?, and Sole of the Little Feet	Business-as-usual	Teacher in classroom	~2 years (with four waves of data collection)	Meditation and mindfulness-based activities incorporated into the school day at teacher’s discretion	Emotion regulation: Emotion Regulation Checklist (ERC) Prosocial skills: Modified Professional Behavioral Questionnaire (Mod-PBQ) General social-emotional functioning: Korean Personality Rating Scale for Children (KPRC)	Guided meditation + mindfulness-based activities resulted in significantly higher scores on lability/negativity, resilience, and prosocial behaviors at the second and third post-intervention assessments. There was no statistically significant difference between groups on adaptive regulation.	High
Lemberger-Truelove 2018 [37]	Complex: 10 min SEL group kindness song and MBI breathing and movement activity, 20 min didactic instruction on SEL/MBI skill or practice, 10 min counselor encouraging participants to vocalize how they might apply the lesson, finally MBI breathing and movement activity	Business-as-usual	Trained counselor in classroom	8 weeks (48 sessions)	4 times a week, 40 min per session	Prosocial skills: inCLASS (Individualized Classroom Assessment Scoring System) Behavioral self-regulation: Child Observation Mindfulness Measure (C-OMM)	Mindfulness has a significant impact on self-regulatory outcomes such as task orientation and orientation to experience. There were no statistically significant results for measures of peer interaction, self-regulated attention, or teacher interaction.	High
Jackman 2019 [35]	Complex: Seven daily practices: focused meditation, loving-kindness meditation, bell exercises, yoga, gratitude practice, kindness and compassion reporting, and feelings finder practices + supplemental learning activities for promoting prosocial behavior	Post-test	Teacher in classroom	1 school year	Daily	Behavioral self-regulation: Head-Toes-Knees-Shoulders (HTKS) Executive Function: Behavior Rating Inventory of Executive Function–Preschool (BREIF-P), Go/No-Go	Children in both groups showed improved scores over time on the HTKS, go/no-go tasks, and meta-cognition. There were no changes in inhibitory self-control summary scale or the total GEC scale. Compared to the control group, children in the OM group performed better on the HTKS and showed decreased cognitive flexibility. There were no differences between the groups on the go/no-go task.	High
Flook 2015 [34]	Complex: Mindfulness-based prosocial skills training aimed at cultivating attention and emotion regulation, with a shared emphasis on kindness practices (e.g., empathy, gratitude, sharing)	Wait-list	Experienced mindfulness instructors in classroom	12 weeks (10 h of training total)	Twice a week, 20–30 min per session	Behavioral self-regulation: Delay of Gratification Task Executive Function: Dimensional Change Card Sort Task (DCCS), Sharing Task Prosocial skills: Teacher-Rated Social Competence (TSC)	The KC intervention group showed greater improvements in social competence and earned higher report card grades in domains of learning, health, and social emotional development, whereas the control group exhibited more selfish behavior over time. Effect sizes favored the KC group on measures of cognitive flexibility and delay of gratification.	Some concerns
Carrozza 2019 [32]	Complex: As part of a mindfulness bibliotherapy intervention, teachers read books like “Visiting Feelings” “Peaceful Piggy Meditation” and used accompanying activities when provided. Teachers utilized lesson plans related to emotions as well as mindfulness.	Bibliotherapy	Teacher in classroom	3 weeks (9 days total)	3 days a week	Emotion regulation: Emotion Regulation Checklist (ERC) General Social-Emotional Functioning: Devereux Early Childhood Assessment for Preschoolers (DECA)	Mindfulness and bibliotherapy used in combination did not produces any effect on child outcomes	Serious *

Note. * assessed by ROBINS-I. SEL = social emotional learning, SSIS-RS = Social Skills Improvement System-Rating Scales, ANT = Attention Network Task, GLT = Global—Local Test, HTKS = Head-Toes-Knees-Shoulders, SDQ = Strengths and Difficulties Questionnaire, BRIEF-P = Behavior Rating Inventory of Executive Function-Preschool, CUMANIN = Children Neuropsychological Maturity Questionnaire, BASC-2 = Behavioral Assessment System for Children, ADHD = attention-deficit/hyperactivity disorder, NEP = SY = Developmental Neuropsy-chological Assessment, CTRS-R:S = Conners Teacher Rating Scale‚ Revised: Short Form, AST = Attention Sustained task, CBQ = Children Behavior Questionnaire, MEFS = Minnesota Executive Func-tion Scale, CBRS = Child Behavior Rating Scale, ERC = Emotion Regulation Checklist, Mod-PBQ = Prosocial skills: Modified Pro-fessional Behavioral Ques-tionnaire, KPRC = Korean Personal-ity Rating Scale for Children, C-OMM = Child Observation Mindful-ness Measure, DCCS = Dimensional Change Card Sort Task, TSC = Teacher-Rated Social Competence, DECA = Devereux Early Childhood Assessment for Preschoolers.

## Data Availability

Not applicable.

## Supplementary Materials

The following are available online at https://www.mdpi.com/article/10.3390/ijerph18116091/s1, Supplementary Materials S1: Search Strategy Sample; Figure S1: Risk of bias for RCT; Figure S2: Risk of bias for Non-RCT; Figure S3: Risk of bias summary for RCT; Figure S4: Risk of bias summary for Non-RCT.

## Appendix A

Quality Appraisal.

**Table A1:** RoB 2 for RCT studies.

RoB 2 for RCT Studies (*n* = 10)						
	1. Risk of Bias Arising from the Randomization Process	2. Risk of Bias Due to Deviations from the Intended Interventions	3. Missing Outcome Data	4. Risk of Bias in Measurement of the Outcome	5. Risk of Bias in Selection of the Reported Result	Overall Risk of Bias
Lim and Qu, 2017 [46]	Some concerns	High	High	Low	Some concerns	High
Kim et al., 2020 [36]	High	Some concerns	Low	High	Some concerns	High
Zelazo et al., 2018 [45]	Low	Some concerns	Low	Some concerns	Some concerns	Some concerns
Jarraya et al., 2019 [15]	Low	Some concerns	Low	Low	Some concerns	Some concerns
Cohen et al., 2018 [33]	Some concerns	High	Low	High	Some concerns	High
Lemberger-Truelove et al., 2018 [37]	Some concerns	High	Low	Low	Some concerns	High
Viglas and Perlman, 2018 [44]	High	Low	Low	High	Some concerns	High
Razza et al., 2020 [40]	Some concerns	Low	Low	Low	Some concerns	Some concerns
Jackman et al., 2019 [35]	Low	Some concerns	Low	High	Some concerns	High
Flook et al., 2015 [34]	Some concerns	Some concerns	Low	Low	Some concerns	Some concerns

**Table A2:** Robins-I for non-RCT studies.

Robins-I for Non-RCT Studies (*n* = 6)								
	1. Bias Due to Confounding	2. Bias in Selection of Participants into the Study	3. Bias in Classification of Interventions	4. Bias Due to Deviations from Intended Interventions	5. Bias Due to Missing Data	6. Bias in Measurement of Outcomes	7. Bias in Selection of the Reported Result	Overall Risk of Bias
Thierry et al., 2018 [43]	Low	Low	Low	Low	Moderate	Low	Low	Moderate
Razza et al., 2015 [39]	Low	Low	Low	Low	Moderate	Low	Low	Moderate
Thierry et al., 2016 [42]	Moderate	Moderate	Low	Low	Low	Serious	Moderate	Serious
Carrozza 2019 [32]	Low	Low	Low	Low	Serious	Serious	NI	Serious
Rich 2010 [41]	Moderate	Low	Low	Critical	Serious	Serious	NI	Critical
Moreno-Gómez and Cejudo, 2019 [38]	Moderate	Low	Low	Low	Low	Moderate	Moderate	Moderate
