# Supplementary material for: Yoga and Mindfulness Interventions for Preschool-Aged Children in Educational Settings: A Systematic Review
Source: Int J Environ Res Public Health. 2021 Jun 5;18(11):6091. doi: 10.3390/ijerph18116091 (PMC8201280; doi:10.3390/ijerph18116091)
Supplement: Supplementary file 1 [file ijerph-18-06091-s001.zip › ijerph-1222517-supplementary/supplementary materials/Search Strategy Sample.pdf]

Supplementary Material Appendix S1

Bibliographic Literature Searching

Database	Hits
PubMed	255
EMBASE	317
PsychInfo	368
ERIC	207
Cochrane Central Register of Controlled Trials	51
American Mindfulness Research Association	294
Total	1492

Database: PubMed
Host: PubMed
Date Searched: May-13-2020
Searcher: Yaoyao Sun

Search Strategy:

Search Number	Strategy	Records Retrieved	Number combi-nation
1	"Yoga"[MeSH Terms]	2772	
2	"yoga"[Title/Abstract]	4633	
3	"yoga"[Other Term]	1388	
4	"Yoga"[MeSH Terms] OR "yoga"[Title/Abstract]	5102	1 OR 2
5	"Mindfulness"[MeSH Terms]	3071	
6	"mindful*"[Title/Abstract]	9693	
7	"mindful*"[Other Term]	3088	
8	compassion[MeSH Terms]	0	
9	"Self-compassion"[MeSH Terms]	0	
10	"self-compassion"[Title/Abstract]	944	
11	"self-compassion"[Other Term]	412	
12	"mind-body therapies"[MeSH Terms]	50045	
13	"Mindfulness"[MeSH Terms] OR "mindful*"[Title/Abstract] OR "self-compassion"[Title/Abstract]	10507	5 OR 6 OR 10
14	"Meditation"[MeSH Terms]	2790	
15	"meditat*"[Title/Abstract]	6068	
16	"meditat*"[Other Term]	1141	

17	contemplation[MeSH Terms]	0	
18	"contemplat*"[Title/Abstract]	7800	
19	"contemplative"[Title/Abstract]	354	
20	"contemplative"[Other Term]	51	
21	"contemplation*"[Title/Abstract]	1446	
22	"contemplation*"[Other Term]	14	
23	"Meditation"[MeSH Terms] OR "meditat*"[Title/Abstract] OR "contemplative"[Title/Abstract] OR "contemplation*"[Title/Abstract]	8582	14 OR 15 OR 19 OR 21
24	"Yoga"[MeSH Terms] OR "yoga"[Title/Abstract] OR "Mindfulness"[MeSH Terms] OR "mindful*"[Title/Abstract] OR "self-compassion"[Title/Abstract] OR "Meditation"[MeSH Terms] OR "meditat*"[Title/Abstract] OR "contemplative"[Title/Abstract] OR "contemplation*"[Title/Abstract]	20363	4 OR 13 OR 23
25	"child, preschool"[MeSH Terms]	908054	
26	"preschool*"[Title/Abstract]	32984	
27	"pre-school*"[Title/Abstract]	4976	
28	"child, preschool"[MeSH Terms] OR "preschool*"[Title/Abstract] OR "pre-school*"[Title/Abstract]	915527	
29	childhood[MeSH Terms]	0	
30	"early child*"[Title/Abstract]	27157	
31	kindergarten[MeSH Terms]	0	
32	"kindergar*"[Title/Abstract]	6680	
33	"Child Care"[MeSH Terms]	19506	
34	child* care[Title/Abstract]	238670	
35	"child care"[Title/Abstract]	7152	
36	"children care"[Title/Abstract]	119	
37	"child day care"[Title/Abstract]	296	
38	"children day care"[Title/Abstract]	27	
39	"child daycare"[Title/Abstract]	106	
40	children daycare[Title/Abstract]	2518	
41	"Child Care"[MeSH Terms] OR "Child Care"[Title/Abstract] OR "children care"[Title/Abstract] OR "child day care"[Title/Abstract] OR "children day	25119	

	care"[Title/Abstract] OR "child daycare"[Title/Abstract]		
42	"schools, nursery"[MeSH Terms]	1461	
43	"nursery school*"[Title/Abstract]	1082	
44	"schools, nursery"[MeSH Terms] OR "nursery school*"[Title/Abstract]	2124	
45	"child, preschool"[MeSH Terms] OR "preschool*"[Title/Abstract] OR "pre-school*"[Title/Abstract] OR "early child*"[Title/Abstract] OR "kindergar*"[Title/Abstract] OR "Child Care"[MeSH Terms] OR "Child Care"[Title/Abstract] OR "children care"[Title/Abstract] OR "child day care"[Title/Abstract] OR "children day care"[Title/Abstract] OR "child daycare"[Title/Abstract] OR "schools, nursery"[MeSH Terms] OR "nursery school*"[Title/Abstract]	951735	28 OR 30 OR 32 OR 41 OR 44
46	("Yoga"[MeSH Terms] OR "yoga"[Title/Abstract] OR "Mindfulness"[MeSH Terms] OR "mindful*"[Title/Abstract] OR "self-compassion"[Title/Abstract] OR "Meditation"[MeSH Terms] OR "meditat*"[Title/Abstract] OR "contemplative"[Title/Abstract] OR "contemplation*"[Title/Abstract]) AND ((((("child, preschool"[MeSH Terms] OR "preschool*"[Title/Abstract] OR "pre-school*"[Title/Abstract]) OR ("early child*"[Title/Abstract])) OR ("kindergar*"[Title/Abstract])) OR ("Child Care"[MeSH Terms] OR "Child Care"[Title/Abstract] OR "children care"[Title/Abstract] OR "child day care"[Title/Abstract] OR "children day care"[Title/Abstract] OR "child daycare"[Title/Abstract])) OR	255	24 AND 45

	("schools, nursery"[MeSH Terms] OR "nursery school*"[Title/Abstract]))		

Database: EMBASE
Host: EMBASE
Date Searched: May-13-2020
Searcher: Yaoyao Sun

Search Strategy:

**Search Number**	**Strategy**	**Records Retrieved**	**Number in website**
1	‘yoga’/exp	7572	
2	yoga:ti,ab,kw	6785	
3	‘yoga’/exp OR yoga:ti,ab,kw	9221	1 OR 2
4	'mindfulness'/exp	8045	
5	mindful*:ti,ab,kw	13045	
6	'compassion'/exp	137	
7	compassion:ti,ab,kw	6879	
8	'self compassion'/exp	156	
9	'self compassion':ti,ab,kw	1067	
10	('compassion'/exp OR compassion:ti,ab,kw) NOT ('self compassion'/exp OR 'self compassion':ti,ab,kw)	5835	
11	'mindfulness'/exp OR mindful*:ti,ab,kw OR 'self compassion'/exp OR 'self compassion':ti,ab,kw	15068	4 OR 5 OR 8 OR 9
12	'meditation'/exp	7872	
13	meditat*:ti,ab,kw	8547	
14	contemplat*:ti,ab,kw	10359	
15	contemplative:ti,ab,kw	387	
16	contemplation*:ti,ab,kw	1875	
17	contemplat*:ti,ab,kw NOT (contemplative:ti,ab,kw OR contemplation*:ti,ab,kw)	8122	
18	'meditation'/exp OR meditat*:ti,ab,kw OR contemplative:ti,ab,kw OR contemplation*:ti,ab,kw	13269	20 OR 21 OR 24 OR 25
19	‘yoga’/exp OR yoga:ti,ab,kw OR 'mindfulness'/exp OR	31256	3 OR 11 OR 18

	mindful*:ti,ab,kw OR 'self compassion'/exp OR 'self compassion':ti,ab,kw OR 'meditation'/exp OR meditat*:ti,ab,kw OR contemplative:ti,ab,kw OR contemplation*:ti,ab,kw		
20	'preschool child'/exp	616772	
21	preschool*:ti,ab,kw	35667	
22	'pre-school*':ti,ab,kw	6829	
23	'pre-school*':ti,ab,kw NOT ('preschool child'/exp OR preschool*:ab,ti,kw)	2196	
24	'preschool-child'/exp OR preschool*:ab,ti,kw OR 'pre-school*':ab,ti,kw	629999	
25	'early childhood'/exp	22	
26	'early child*':ti,ab,kw	34701	
27	'child care'/exp	52341	
28	'child* care':ti,ab,kw	8042	
29	'child* day care':ti,ab,kw	423	
30	'child* daycare':ti,ab,kw	83	
31	'child* daycare':ti,ab,kw NOT 'child* day care':ti,ab,kw	58	
32	'child care'/exp OR 'child* care':ti,ab,kw OR 'child* day care':ti,ab,kw OR 'child* daycare':ti,ab,kw	57158	
33	'kindergarten'/exp	2739	
34	kindergar*:ti,ab,kw	7800	
35	'nursery school'/exp	1483	
36	'nursery school*':ti,ab,kw	1395	
37	'nursery school'/exp OR 'nursery school*':ab,ti,kw	2318	
38	'preschool child'/exp OR preschool*:ab,ti,kw OR 'pre-school*':ab,ti,kw OR 'early child*':ti,ab,kw OR 'child care'/exp OR 'child* care':ti,ab,kw OR 'child* day care':ti,ab,kw OR 'child* daycare':ti,ab,kw OR kindergar*:ti,ab,kw OR 'nursery school'/exp OR 'nursery school*':ab,ti,kw	702413	24 OR 26 OR 32 OR 34 OR 37

51	(‘yoga’/exp OR yoga:ti,ab,kw OR 'mindfulness'/exp OR mindful*:ti,ab,kw OR 'self compassion'/exp OR 'self compassion':ti,ab,kw OR 'meditation'/exp OR meditat*:ti,ab,kw OR contemplative:ti,ab,kw OR contemplation*:ti,ab,kw) AND ('preschool child'/exp OR preschool*:ab,ti,kw OR 'pre-school*':ab,ti,kw OR 'early child*':ti,ab,kw OR 'child care'/exp OR 'child* care':ti,ab,kw OR 'child* day care':ti,ab,kw OR 'child* daycare':ti,ab,kw OR kindergar*:ti,ab,kw OR 'nursery school'/exp OR 'nursery school*':ab,ti,kw)	317	19 AND 38

Database: PsychInfo
Host: EBSCO
Date Searched: May-13-2020
Searcher: Renee Lamoreau

Search Strategy:

**Search Number**	**Strategy**	**Records Retrieved**	**Number in website**
1	SU Yoga	2,069	S1
2	TI Yoga OR AB Yoga	2,726	S2
3	SU Yoga OR TI Yoga OR AB Yoga	**2,961**	S3
4	TI Yoga or AB Yoga NOT SU Yoga	884	S4
5	SU Yoga NOT TI/AB Yoga	227	S5
6	KW Yoga	1,731	S6

7	KW Yoga OR SU Yoga OR AB Yoga OR TI Yoga	2,953	S7
8	KW Yoga NOT (SU Yoga OR AB Yoga OR TI Yoga)	0	S8
9	SU mindful*	10,996	S9
10	TI mindful*	8,468	S10
11	AB mindful*	14,881	S11
12	SU Mindful* OR TI mindful* OR AB mindful*	15,828	S12
13	KW mindful*	10,154	S13
14	KW mindful* NOT (SU Mindfulness OR TI mindful* OR AB mindful*)	4	S14
15	SU self-compassion	1,153	S15
16	TI self-compassion	945	S16
17	AB self-compassion	1,666	S17
18	SU self-compassion OR TI self-compassion OR AB self-compassion	1,726	S18
19	SU meditat*	5,507	S19
20	TI meditat*	3,126	S20
21	AB meditat*	8,325	S21
22	SU meditat* OR TI meditat* OR AB meditat*	9,642	S22
23	TI contemplat*	530	S23

24	TI contemplat* OR AB contemplat* OR KW Contemplat*	6,814	S24
25	SU Preschool* OR SU Pre-School* OR TI Preschool* OR AB Preschool* OR TI Pre-School* or AB Pre-School*	103,295	S25
26	SU Kindergar* OR TI Kindergar* OR AB Kindergar*	21,379	S26
27	SU "Early childhood" OR TI "Early childhood" OR AB "Early childhood"	32,419	S27
28	SU (Daycare OR "Day Care") OR TI (Daycare OR "Day Care") OR AB (Daycare OR "Day Care")	8,205	S28
29	SU (Childcare OR "Child Care") OR TI (Childcare OR "Child Care") OR AB (Childcare OR "Child Care")	19,919	S29
32	SU ("nursery school*" OR "nursery school students") OR TI ("nursery school*" OR "nursery school students") OR AB ("nursery school*" OR "nursery school students")	2,960	S32
30	S25 OR S26 OR S27 OR S28 OR S29	159,212	S30
31	FINAL SEARCH<br><br>S30 AND (S3 OR S12 OR S18 OR S22 OR S24)	**368**	S31

Database: ERIC
Host: EBSCO

Date Searched: May-13-2020
Searcher: Renee Lamoreau

Search Strategy:

**Search Number**	**Strategy**	**Records Retrieved**	**Number in website**
1	SU Yoga	0	S1
2	TI Yoga OR AB Yoga	252	S2
3	KW Yoga	41	S3
4	TI Yoga OR AB Yoga OR KW YOGA	259	S4
5	SU Mindfulness	0	S5
6	TI mindful*	715	S6
7	AB mindful*	1,686	S7
8	KW mindful* OR TI mindful* OR AB mindful*	1,741	S8
9	KW mindful*	6	S9
10	KW self-compassion	0	S10
11	TI self-compassion	62	S11
12	AB self-compassion	97	S12
13	TI self-compassion OR AB self-compassion	97	S13
14	SU meditation OR KW meditat*	180	S14
15	TI meditat*	260	S15
16	AB meditat*	781	S16
17	SU meditation OR KW meditat*OR TI	906	S17

	meditat* OR AB meditat*		
18	TI contemplat*	173	S18
19	TI contemplat* OR AB contemplat* OR KW contemplat*	1,886	S19
20	SU Preschool OR TI Preschool OR AB Preschool	50,013	S20
21	SU Kindergar* OR TI Kindergar* OR AB Kindergar*	25,224	S21
22	SU "Early child*" OR TI "Early child*" OR AB "Early child*"	55,435	S22
23	SU (Daycare OR "Day Care") OR TI (Daycare OR "Day Care") OR AB (Daycare OR "Day Care")	10,449	S23
24	SU (Childcare OR "Child Care") OR TI (Childcare OR "Child Care") OR AB (Childcare OR "Child Care")	14,961	S24
25	S20 OR S21 OR S22 OR S23 OR S24	106,930	S25
26	FINAL SEARCH<br><br>S25 AND (S3 OR S12 OR S18 OR S22 OR S24)	207	S26

Database: Cochrane
Host: Cochrane Library
Date Searched: Apr-14-2020
Searcher: Raquel Horlick

Search Strategy:

Search Number	Strategy	Records Retrieved	Number combi-nation
1	"Yoga"[MeSH Terms]	633	
2	(yoga):ti,ab,kw	3026	
3	"Yoga"[MeSH Terms] NOT (yoga):ti,ab,kw	0	
4	(yoga):ti,ab,kw	3026	2
5	"Mindfulness"[MeSH Terms]	749	
6	(mindful*):ti,ab,kw	4862	
7	"Mindfulness"[MeSH Terms] NOT (mindful*):ti,ab,kw	0	
8	("self compassion"):ti,ab,kw	431	
9	(mindful*):ti,ab,kw OR ("self compassion"):ti,ab,kw	5012	6 OR 8
10	"Meditation"[MeSH Terms]	575	
11	(mediat*):ti,ab,kw	34851	
12	(meditation):ti,ab,kw	2569	
13	(contemplation*):ti,ab,kw	275	
14	(contemplative):ti,ab,kw	59	
15	(meditation):ti,ab,kw OR (contemplation*):ti,ab,kw OR (contemplative):ti,ab,kw	2876	12 OR 13 OR 14
16	(yoga):ti,ab,kw OR (mindful*):ti,ab,kw OR ("self compassion"):ti,ab,kw OR (meditation):ti,ab,kw OR (contemplation*):ti,ab,kw OR (contemplative):ti,ab,kw	8842	4 OR 9 OR 15
17	"child, preschool"[MeSH Terms]	1172	
18	(preschool*):ti,ab,kw	36633	
19	(pre-school*):ti,ab,kw	646	
20	(preschool*):ti,ab,kw OR (pre-school*):ti,ab,kw	36641	
21	("early child*"):ti,ab,kw	134	
22	(kindergar*):ti,ab,kw	667	
23	"Child Care"[MeSH Terms]	801	
24	("child care"):ti,ab,kw	1227	
25	("child day care"):ti,ab,kw	250	
26	("child daycare"):ti,ab,kw	10	

27	("child care"):ti,ab,kw OR ("child day care"):ti,ab,kw OR ("child daycare"):ti,ab,kw	1413	
28	"schools, nursery"[MeSH Terms]	37	
29	("nursery school*"):ti,ab,kw	55	
30	(preschool*):ti,ab,kw OR (pre-school*):ti,ab,kw OR ("early child*"):ti,ab,kw OR (kindergar*):ti,ab,kw OR ("child care"):ti,ab,kw OR ("child day care"):ti,ab,kw OR ("child daycare"):ti,ab,kw OR ("nursery school*"):ti,ab,kw	37844	20 OR 21 OR 22 OR 27 OR 29
31	((yoga):ti,ab,kw OR (mindful*):ti,ab,kw OR ("self compassion"):ti,ab,kw OR (meditation):ti,ab,kw OR (contemplation*):ti,ab,kw OR (contemplative):ti,ab,kw) AND ((preschool*):ti,ab,kw OR (pre-school*):ti,ab,kw OR ("early child*"):ti,ab,kw OR (kindergar*):ti,ab,kw OR ("child care"):ti,ab,kw OR ("child day care"):ti,ab,kw OR ("child daycare"):ti,ab,kw OR ("nursery school*"):ti,ab,kw)	51	16 AND 30

The following supplemental searches were undertaken in American Mindfulness Research Association on 04/29/2020.

Retrieved related results.

Search Number	Strategy	Records retrieved	Notes	Chosen Articles
1	Preschool	15	Including “preschools”, “preschooler”, “preschoolers”.	
2	Pre-school	1		
3	child	269	Including “children”,	
4	kindergar	2		
5	nursery	0		
6	kid	7		
