# Supplementary figures and images for: Yoga and Mindfulness Interventions for Preschool-Aged Children in Educational Settings: A Systematic Review
Source: Int J Environ Res Public Health. 2021 Jun 5;18(11):6091. doi: 10.3390/ijerph18116091 (PMC8201280; doi:10.3390/ijerph18116091)

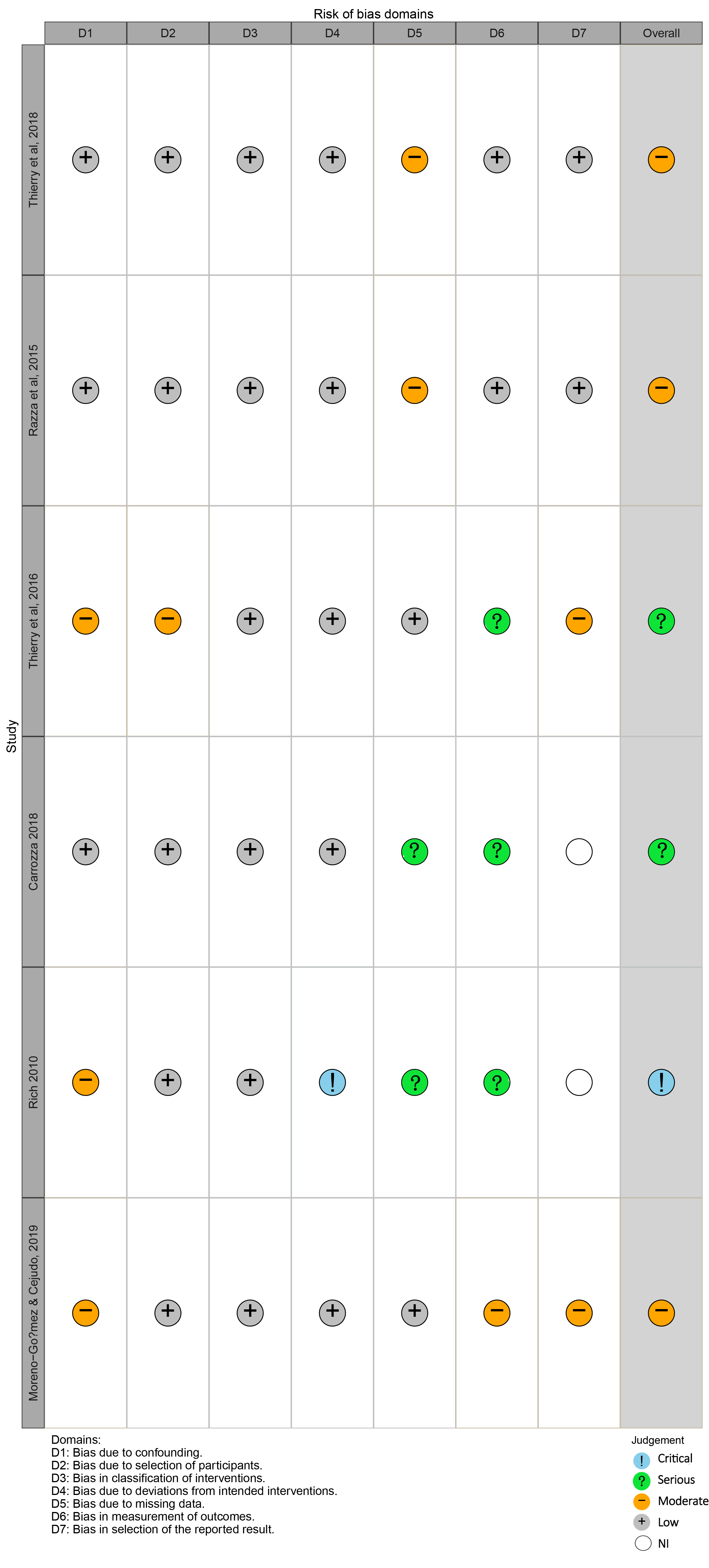

Supplement: Supplementary file 1 [file ijerph-18-06091-s001.zip › ijerph-1222517-supplementary/supplementary materials/Figure S2.jpg]

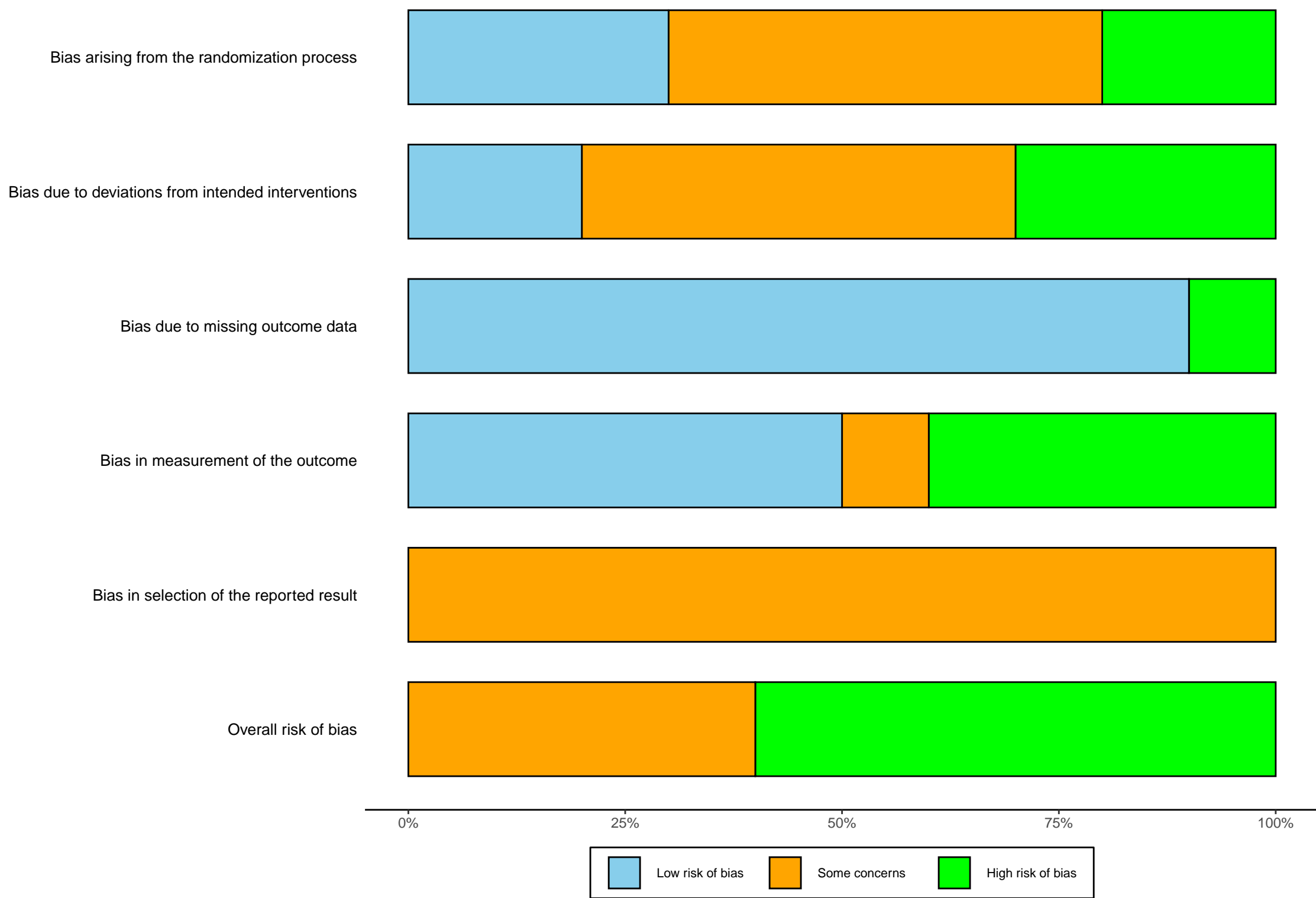

Supplement: Supplementary file 1 [file ijerph-18-06091-s001.zip › ijerph-1222517-supplementary/supplementary materials/Figure S3.pdf]

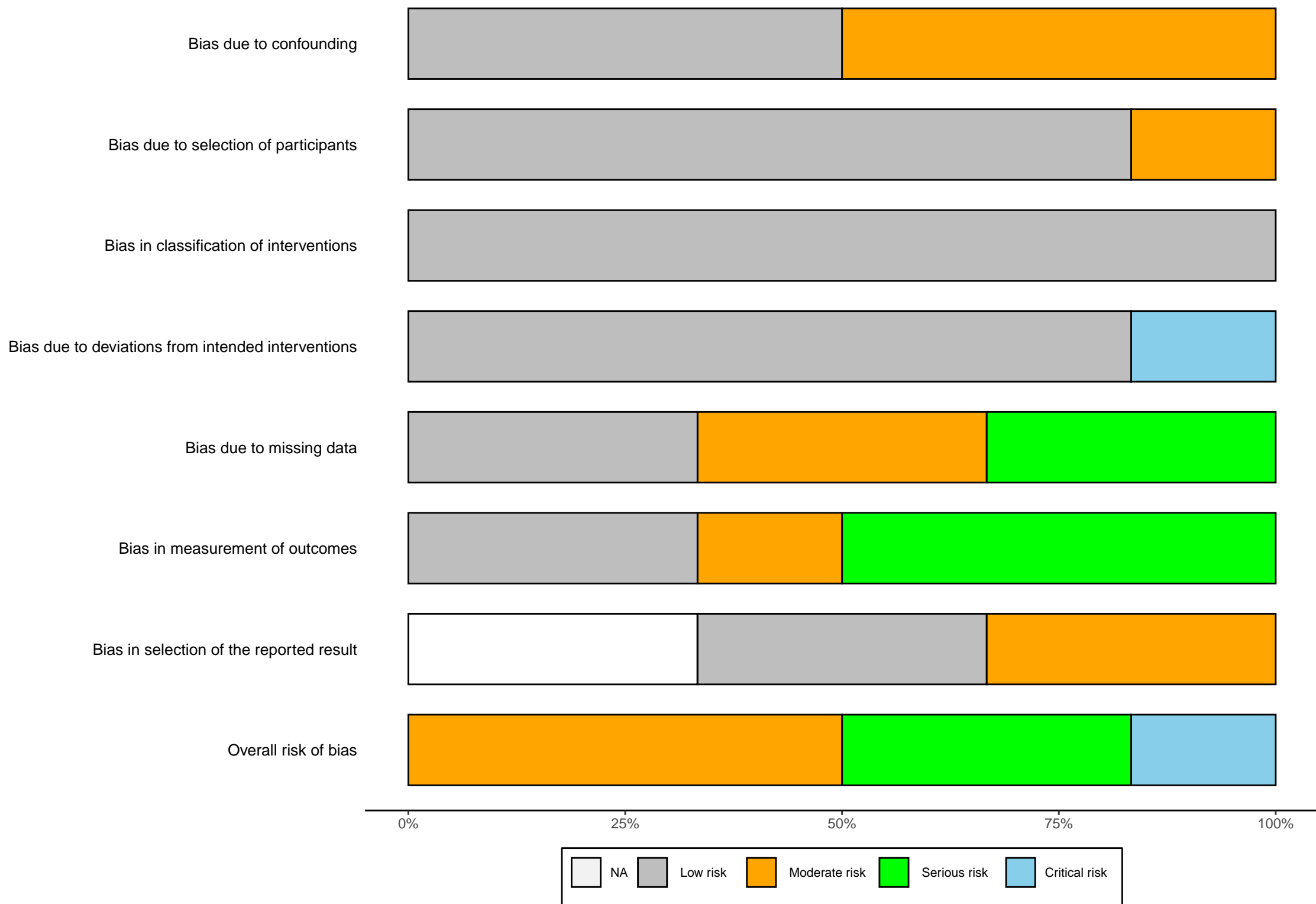

Supplement: Supplementary file 1 [file ijerph-18-06091-s001.zip › ijerph-1222517-supplementary/supplementary materials/Figure S4.pdf]
