# Supplementary material for: Yoga and Mindfulness Interventions for Preschool-Aged Children in Educational Settings: A Systematic Review
Source: Int J Environ Res Public Health. 2021 Jun 5;18(11):6091. doi: 10.3390/ijerph18116091 (PMC8201280; doi:10.3390/ijerph18116091)
Supplement: Supplementary file 1 [file Figure S1.pdf › ijerph-1222517-supplementary/supplementary materials/Figure S1.pdf]

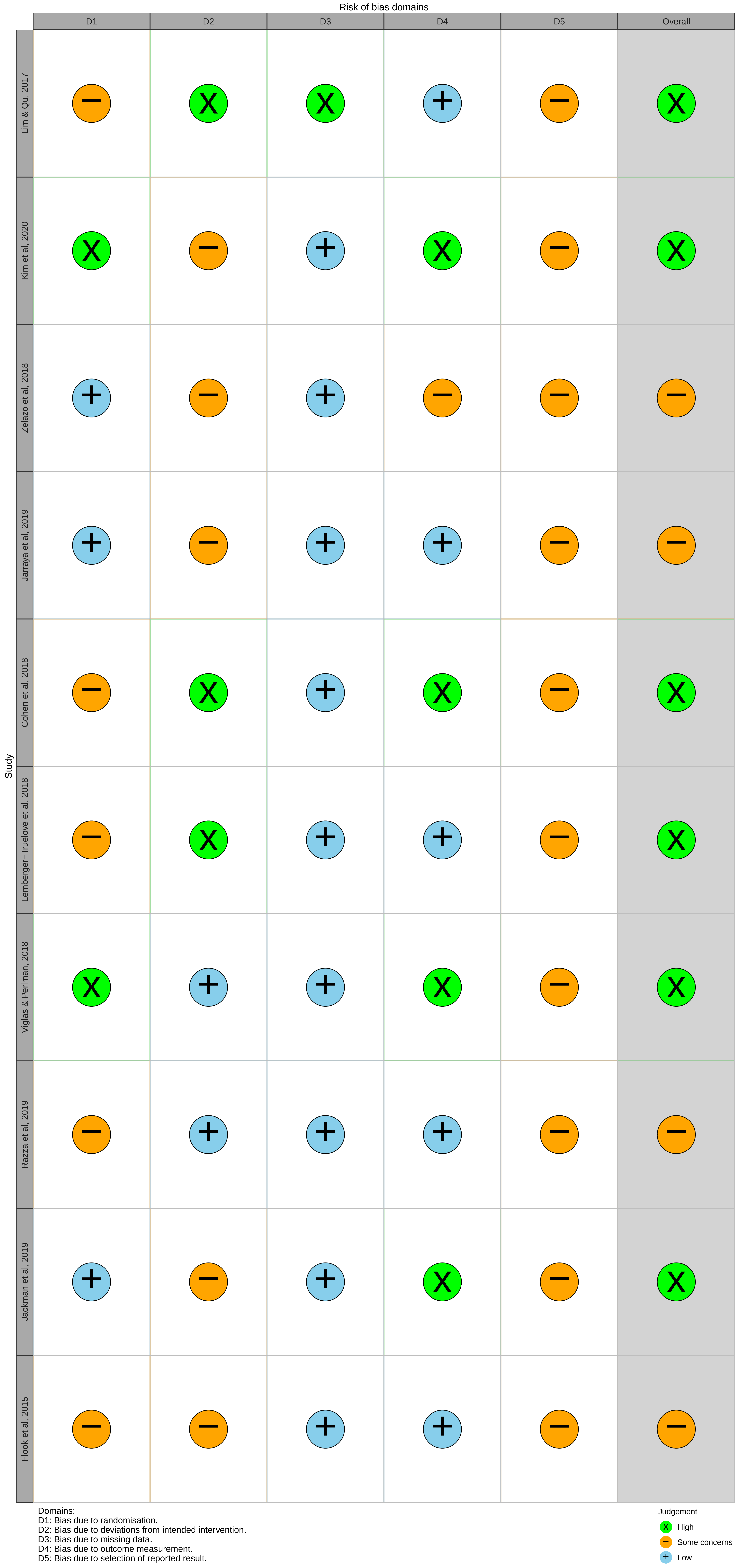
Risk of bias domains
D1
D2
D3
D4
D5
Overall
Study
Lim & Qu, 2017
Kim et al, 2020
Zelazo et al, 2018
Jarraya et al, 2019
Cohen et al, 2018
Lemberger–Truelove et al, 2018
Viglas & Perlman, 2018
Razza et al, 2019
Jackman et al, 2019
Flook et al, 2015
Domains:
D1: Bias due to randomisation.
D2: Bias due to deviations from intended intervention.
D3: Bias due to missing data.
D4: Bias due to outcome measurement.
D5: Bias due to selection of reported result.
Judgement
High
Some concerns
Low
